# Delayed decision, harder landing: how mental fatigue alters reactive step biomechanics and joint loading in table tennis players

**DOI:** 10.3389/fpsyg.2026.1871714

**Published:** 2026-10-01

**Authors:** Qiang Li, Lingyue Meng, Yubo Wang, Yibo Wang, Zhekun Yang

**Affiliations:** 1 School of Sports Law, Shanghai University of Political Science and Law, Shanghai, China; 2 Physical Education and Sports School, Soochow University, Suzhou, Jiangsu, China; 3 Rehabilitation Engineering Lab, Department of Kinesiology and Community Health, University of Illinois at Urbana-Champaign, Champaign, IL, United States; 4 School of Law, Anhui Medical University, Hefei, Anhui, China

**Keywords:** joint loading, lower-limb biomechanics, mental fatigue, reactive stepping, table tennis

## Abstract

**Objective:**

Mental fatigue (MF) is known to impair psychobiological performance, yet its cross-dimensional impact on the neuromechanical control of reactive athletic movements remains underexplored. This study investigated the effects of MF on the cognitive-temporal and lower-limb biomechanical characteristics of table tennis athletes executing a reactive chasse-step.

**Methods:**

Twenty-four collegiate table tennis athletes (12 males, 12 females) completed a randomized, counterbalanced crossover study. Participants performed a choice-reaction chasse-step forehand topspin task following either a 45-min stroop task (MF condition) or neutral documentary viewing (Control condition). Three-dimensional motion capture and force plates were synchronized to analyze cognitive-motor timing and lower-limb biomechanics across the initial landing, propulsion, and stabilization phases.

**Results:**

The Stroop task successfully induced significant subjective mental fatigue (*d* = 9.03). Behaviorally, MF was associated with a cognitive-motor time compression effect, significantly prolonging premotor time (*d* = 4.26) while reducing chasse-step completion time (*d* = 6.03). Biomechanically, the MF condition was characterized by a shift toward a “stiff landing” strategy, decreasing maximum ankle dorsiflexion during initial landing (*d* = 0.57) and minimum knee flexion during stabilization (*d* = 0.72). Kinetically, this reduced shock absorption was accompanied by substantial increases in peak vertical ground reaction force (*d* = 0.75), loading rate (*d* = 0.97), and medial-lateral ground reaction force (*d* = 0.93). Additionally, peak knee extension moments and proximal joint extension velocities were significantly reduced during the propulsion phase.

**Conclusion:**

MF is linked to a cognitive delay and motor compression cascade that alters the temporal structure of reactive footwork. This central resource depletion may lead athletes to adopt a rigid landing strategy, sacrificing shock absorption and propulsive efficiency. Consequently, MF is associated with elevated multi-planar joint impact forces, highlighting it as a potential biomechanical risk factor for lower-limb injuries. Ultimately, athletes’ cognitive state should be integrated into routine monitoring to balance performance optimization and injury prevention in high-speed racket sports.

## 1 Introduction

Mental fatigue (MF) is a psychobiological state induced by prolonged or high-intensity cognitive exertion, primarily characterized by an increased subjective sense of exhaustion alongside an objective decline in both cognitive and motor performance (Marcora et al., 2009; Russell et al., 2019; Goodman et al., 2025). In recent years, the negative impact of MF on sports performance has garnered widespread attention (Pan et al., 2024; Pan et al., 2025). Research indicates that MF not only impairs core cognitive functions, such as attention, reaction speed, and decision-making (Sun et al., 2021), but also deteriorates the quality of subsequent motor task execution by elevating the rating of perceived exertion (RPE) (Pitts and Bhatt, 2023; Pageaux and Lepers, 2018).

This effect is particularly pronounced in racket sports, which heavily rely on sustained attention and rapid visual information processing. For instance, Mansec et al. (2018) found that after enduring 90 min of cognitive load, table tennis players experienced a 2.2% decrease in stroke speed and a 3.9% reduction in accuracy. Habay et al. (2021) further confirmed that MF significantly prolongs the visuomotor reaction time of table tennis players. Similarly, both tennis and badminton players have exhibited marked deterioration in serve accuracy and visuomotor reaction times following mental fatigue (Ding et al., 2024). Collectively, these findings highlight MF as a critical boundary factor limiting performance in racket sports.

As table tennis is a high-speed, competitive sport, its technical execution requires not only rapid cognitive decision-making but also the highly efficient operation of the lower-limb kinetic chain (Iino and Kojima, 2009). In typical match scenarios, athletes must interpret incoming ball information and execute appropriate footwork adjustments to reach the optimal hitting position within extremely short timeframes—often less than 500 milliseconds. The chasse-step serves as the core foundational footwork for forehand attacks in table tennis. Its biomechanical quality directly dictates the momentum generation prior to the stroke and post-stroke postural stability: athletes must achieve rapid weight transfer and dynamic stability by coordinating the lateral drive of the non-dominant foot with the explosive push-off and rotation of the dominant foot (He et al., 2021). However, during high-intensity competitive matches, the execution of this footwork is frequently compounded by the continuous accumulation of cognitive load (Mansec et al., 2018).

Although existing studies have extensively described the kinematic and kinetic characteristics of table tennis footwork (He et al., 2021; Iino, 2018), these investigations have predominantly been conducted under non-fatigued or isolated physically fatigued states. Consequently, they fail to reveal how cognitive exertion (i.e., MF) cross-dimensionally disrupts the biomechanical control of the lower limbs. From the perspective of neuromechanical coupling, MF depletes the brain’s executive control resources, leading to delayed motor decision-making, which manifests as increased reaction times (Habay et al., 2021). This delay at the cognitive level inevitably compresses the available time for the subsequent motor execution phase. Forced to complete the stroke within a shortened time window, athletes are often compelled to make hasty kinematic adjustments (Besier et al., 2001). This can result in diminished neuromuscular control efficiency and the emergence of compensatory motor strategies (Enoka and Duchateau, 2016).

Recent biomechanical research provides indirect evidence for these compensatory strategies. Kong et al. (2023) demonstrated that MF causes patients with functional ankle instability to exhibit reduced ankle stiffness and increased loading rates during unanticipated side-step cutting maneuvers. More critically, studies focusing on drop jump movements have identified a characteristic “MF-induced stiff landing” alteration pattern. Following the induction of MF, subjects generally exhibit significantly reduced ankle dorsiflexion and knee flexion angles at the moment of touchdown, accompanied by substantial increases in peak vertical ground reaction force and knee extension moments (Zhang et al., 2025). This hard landing strategy, characterized by a lack of proper shock absorption, not only reduces the energy transfer efficiency within the lower-limb kinetic chain but also potentially elevates the risk of lower-limb joint injuries.

However, it must be noted that the aforementioned studies investigating the biomechanical impacts of MF almost exclusively employed pre-planned motor tasks. In contrast, during table tennis matches, athletes are confronted with highly “reactive” footwork tasks—meaning they must make real-time movement decisions based on unpredictable external visual stimuli. Henry et al. (2016) pointed out that cognitive decision-making ability is the primary determinant of reactive agility performance, rendering reactive tasks significantly more sensitive to MF than their pre-planned counterparts (Paul et al., 2016; Young et al., 2015). Therefore, during reactive chasse-steps in table tennis, which inherently carry extreme cognitive demands, the kinematic stiffening and kinetic overloading effects induced by MF may be further amplified. Yet, to date, no research has directly quantified the impact of MF on the biomechanical characteristics of reactive footwork in table tennis. This gap in the literature fundamentally limits our mechanistic understanding of how cognitive-motor interactions precipitate lower-limb micro-injuries and power degradation in this sport.

Against this background, the present study utilized a 45-min Stroop color-word interference task to induce MF. By integrating three-dimensional motion capture with force plate technology, this study aimed to investigate the effects of MF on the cognitive-temporal and lower-limb biomechanical characteristics of reactive chasse-step forehand topspin strokes in table tennis players. We propose the following hypotheses: (1) MF will compromise cognitive-motor execution efficiency, manifesting as prolonged reaction and decision-making times (pre-motor time); (2) MF will alter lower-limb biomechanics, leading to stiffer landing and shock absorption characteristics; and (3) MF will induce abnormal joint loading profiles.

## 2 Methods

### 2.1 Participants

An *a priori* power analysis was conducted using G*Power software (version 3.1.9.7) to determine the required sample size. Based on a conservative medium effect size (d_z_ = 0.6) for paired-samples t-tests derived from previous biomechanical crossover studies, an alpha level of 0.05, and a target power of 0.80, the minimum required sample size was 24. Consequently, a total of 24 collegiate table tennis athletes were recruited for this study, ensuring sufficient statistical power. All participants were national second-grade athletes with at least 8 years of formal training experience (11.20 ± 4.10 years). Morphological characteristics were as follows: sex, 12 males, 12 females; age, 20.90 ± 1.70 years; height, 171.90 ± 5.50 cm; and body mass, 66.30 ± 11.90 kg. Inclusion criteria required: (1) no history of lower-limb injuries or surgeries within the previous 12 months; (2) normal or corrected-to-normal vision to ensure accurate identification of visual stimuli; and (3) no known cognitive or neurological disorders. Participants were instructed to abstain from caffeine and strenuous exercise for 24 h prior to testing and to maintain at least 7 h of sleep. The study was approved by the Ethics Committee of Soochow University (SUDA20260331H03), and all participants provided written informed consent.

### 2.2 Experimental design

The study employed a randomized crossover repeated-measures design. Each participant visited the laboratory twice, separated by a washout period of at least 72 h to eliminate carryover effects (Smith et al., 2016). The testing order (Control [CON] vs. Mental Fatigue [MF]) was counterbalanced using a Latin square design. The overall experimental procedure is illustrated in Figure 1.

**Figure 1:**
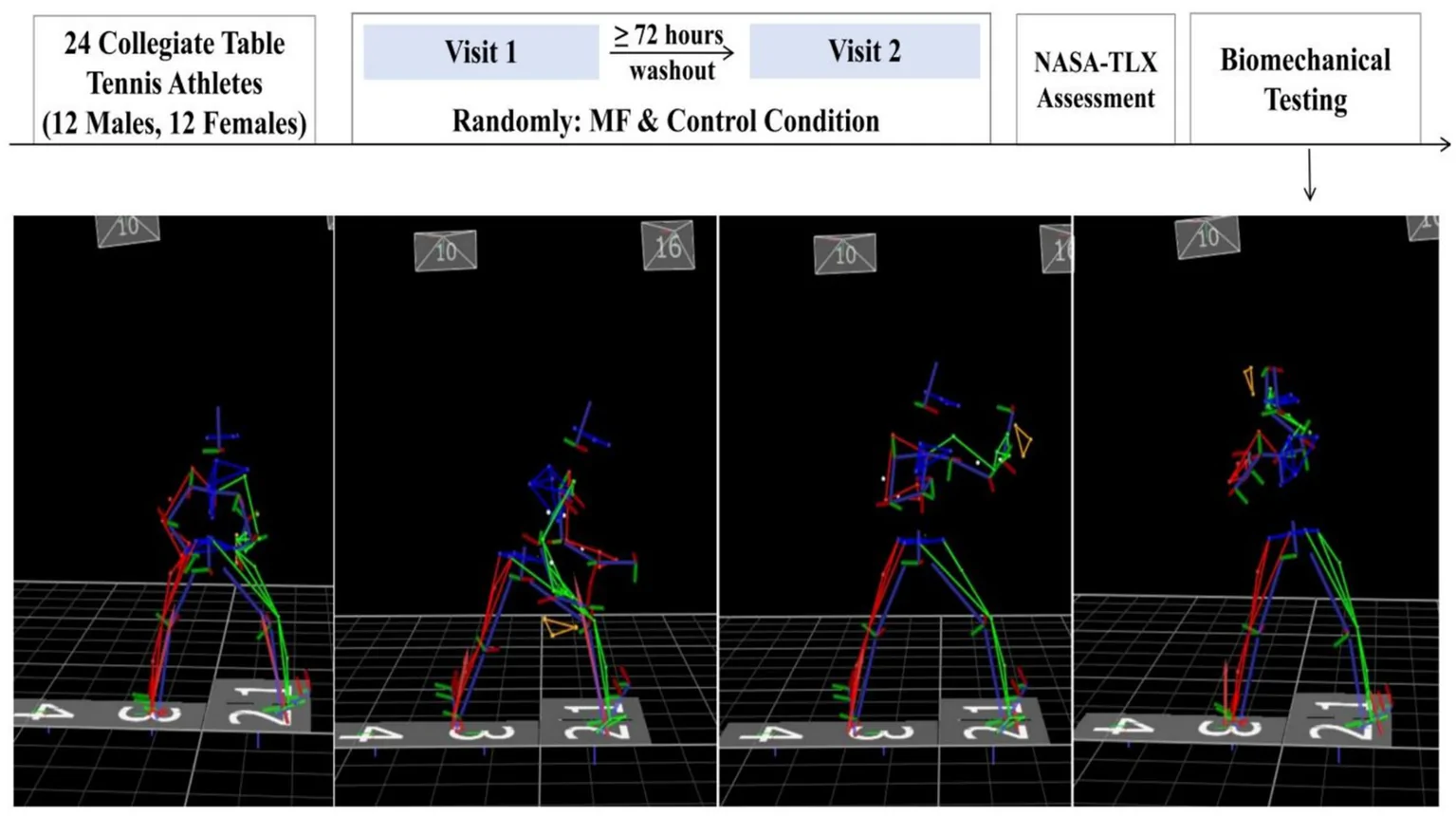
Experimental procedure. MF indicates mental fatigue.

### 2.3 Mental fatigue induction and manipulation check

In the MF condition, participants performed a 45-min computerized version of the Stroop color-word conflict task consisting of 1,350 trials using Python-based Psychopy software (v 2023.2). They were required to respond as quickly and accurately as possible to the font color of the displayed characters, with auditory feedback provided for errors or delayed responses (>1,500 ms) (Pageaux et al., 2013). In the CON condition, participants watched a neutral 45-min documentary in the same environment. This specific 45-min cognitive exertion versus neutral documentary protocol has been widely validated to effectively induce and isolate mental fatigue (Smith et al., 2016; Van Cutsem et al., 2017). To verify the induction effect, subjective fatigue was assessed using the NASA-TLX scale (Hart and Staveland, 1988). Objective cognitive performance was quantified by the “Time-on-Task effect,” comparing the error rate between the first and last 10 min of the Stroop task (Boksem et al., 2005).

### 2.4 Reactive chasse-step task

Within 3 min of completing the fatigue intervention, participants performed a reactive chasse-step forehand looping task. A choice-reaction paradigm was utilized to induce prepotent motor responses (Wessel, 2018): participants maintained a ready stance at the center of the testing area and reacted to randomized visual signals.

Go Signal (70%): A red LED light triggered the ball machine to deliver a ball to the forehand side; participants executed a chasse-step and a maximum-effort forehand loop, ensuring the right foot landed on the target force plates.

No-Go Signal (30%): A green LED light required participants to inhibit the movement and remain in the ready position.

Thirty valid trials (21 Go, 9 No-Go) were recorded for each condition, with 30-s intervals between trials.

### 2.5 Data acquisition and instrumentation

Kinematic data were captured at 100 Hz using a 16-camera Vicon motion capture system (MX13, Oxford Metrics, UK). In accordance with the Vicon Plug-in Gait full body model, 39 circular reflective markers (14 mm diameter) were attached to bony landmarks of the pelvis and lower limbs to ensure accurate kinematic data collection.

Kinetic data were recorded synchronously at 1,000 Hz using four embedded Kistler force plates (9287B, Switzerland). The visual stimulus signal was integrated into the Vicon system via an analog input channel to ensure microsecond-level synchronization for time-latency calculations. After marker placement, a static calibration trial was collected using the Vicon system to verify clear, unobstructed detection of all markers. Formal testing began only after successful calibration.

### 2.6 Data processing and phase definition

Data processing was performed using Vicon Nexus 2.12 and MATLAB R2022a. Both kinematic and kinetic data were smoothed using a fourth-order zero-lag Butterworth low-pass filter with cutoff frequencies of 12 Hz and 50 Hz, respectively (Decker et al., 2003). To account for individual anthropometric differences, ground reaction forces (GRF) and loading rates (LR) were normalized to body weight (BW and BW/s), and internal joint moments were normalized to body mass (N·m/kg). This normalization procedure is grounded in the dynamic similarity hypothesis, which states that mechanical loads must be proportionately scaled to an individual’s body dimensions to eliminate the confounding effects of morphological variance (Hof, 1996; Winter, 2009). Converting absolute force (N) to multiples of BW provides a more universally interpretable metric for evaluating lower-limb joint load (Meng et al., 2022).

The dependent variables were categorized into cognitive-temporal measures and biomechanical characteristics. As detailed: Premotor Time (PMT, ms), defined as the duration from the onset of the visual LED signal to the initiation of movement. Movement initiation was kinematically identified when the lateral horizontal velocity of the pelvis center of mass exceeded 0.05 m/s for a continuous 50 ms. This customized velocity-time threshold was established to effectively filter out inherent postural sway and marker noise prior to the actual voluntary movement initiation (Wheeler and Sayers, 2010). Chasse-step completion time (CCT, ms), defined as the duration from movement initiation to the right foot initial contact (RF_IC) on the target force plate. It is imperative to clarify that CCT operationally isolates the lower-limb spatial repositioning phase. It represents the temporal window required to establish the foundational biomechanical posture prior to the actual racket-ball impact, rather than the full stroke execution cycle; NASA-TLX mental demand score (0–100 points); Stroop error rate (%).

To accurately capture the neuromechanical coupling during the task, the stroke execution cycle—from right foot initial contact (RF_IC) to take-off, which was temporally partitioned into three consecutive functional phases based on synchronized kinematic and kinetic events (Chappell et al., 2002). Given that the right leg acts as the principal load-bearing and propulsive pivot, discrete biomechanical variables were extracted exclusively from the right lower extremity: Phase 1 (P1): defined as the interval from RF_IC (vertical GRF > 50 N) to the instant of peak right knee flexion. Variables extracted included the peak knee flexion and peak ankle dorsiflexion angles (°), the peak vertical GRF (PvGRF, BW), the vertical loading rate (LR, BW/s, calculated as the slope between 20% and 80% of the initial peak force) (Milner et al., 2006), and the peak knee extension moment (N·m/kg). Phase 2 (P2): defined as the interval from peak right knee flexion to peak right knee extension. Variables extracted included the peak extension/plantarflexion angular velocities (°/s) and peak joint moments of the hip, knee, and ankle (N·m/kg). Phase 3 (P3): defined as the interval from peak right knee extension to right foot take-off (vertical GRF < 50 N). Variables extracted included the minimum knee flexion angle (°) and the peak medial-lateral GRF (MLGRF, BW).

### 2.7 Statistical analysis

Data were analyzed using SPSS 26.0 (IBM Corp., Armonk, NY, USA) and presented as mean ± standard deviation (Mean ± SD). Paired-samples *t*-tests were used to evaluate differences between CON and MF conditions for cognitive, behavioral, and biomechanical variables. For paired-samples comparisons, effect sizes were calculated as Cohen’s d using the formula d_z_ = t / $\sqrt{N}$ (Lakens, 2013). It should be noted that because the d_z_ formula utilizes the SD of the difference scores, effectively controlling for between-subject variance, it mathematically generates substantially larger magnitudes than independent-sample formulas. Consequently, the large effect sizes reported in this within-subject design indicate high internal consistency of the intervention rather than absolute magnitude shifts comparable to general population benchmarks. Significance was set at *α* = 0.05. Additionally, to ensure rigorous statistical transparency regarding the multiple independent comparisons made during the exploratory biomechanical profiling, Bonferroni-adjusted *p*-values were calculated for the biomechanical variables and are provided in Supplementary Table S1.

## 3 Results

### 3.1 Verification of mental fatigue induction

To verify the effectiveness of the mental fatigue induction protocol, a combined subjective and objective assessment was adopted in this study. For objective cognitive performance, a paired-samples *t*-test evaluated the impact of the 45-min Stroop color-word conflict task on the error rate. There was a statistically significant increase in the Stroop error rate from the first 10 min to the last 10 min (t_(23)_ = 14.856, *p* < 0.001, *d* = 3.03, shown in Figure 2). For subjective cognitive workload evaluation, the NASA-TLX score in the MF condition (M = 76.63, SD = 6.33) was significantly higher than that in the CON condition (M = 25.83, SD = 2.91, t_(23)_ = 44.229, *p* < 0.001, *d* = 9.03). The exceptionally large effect sizes (*d* > 3.0) in both objective performance and subjective perception confirmed that participants were in a state of profound cognitive exhaustion prior to performing the reactive chasse-step task.

**Figure 2:**
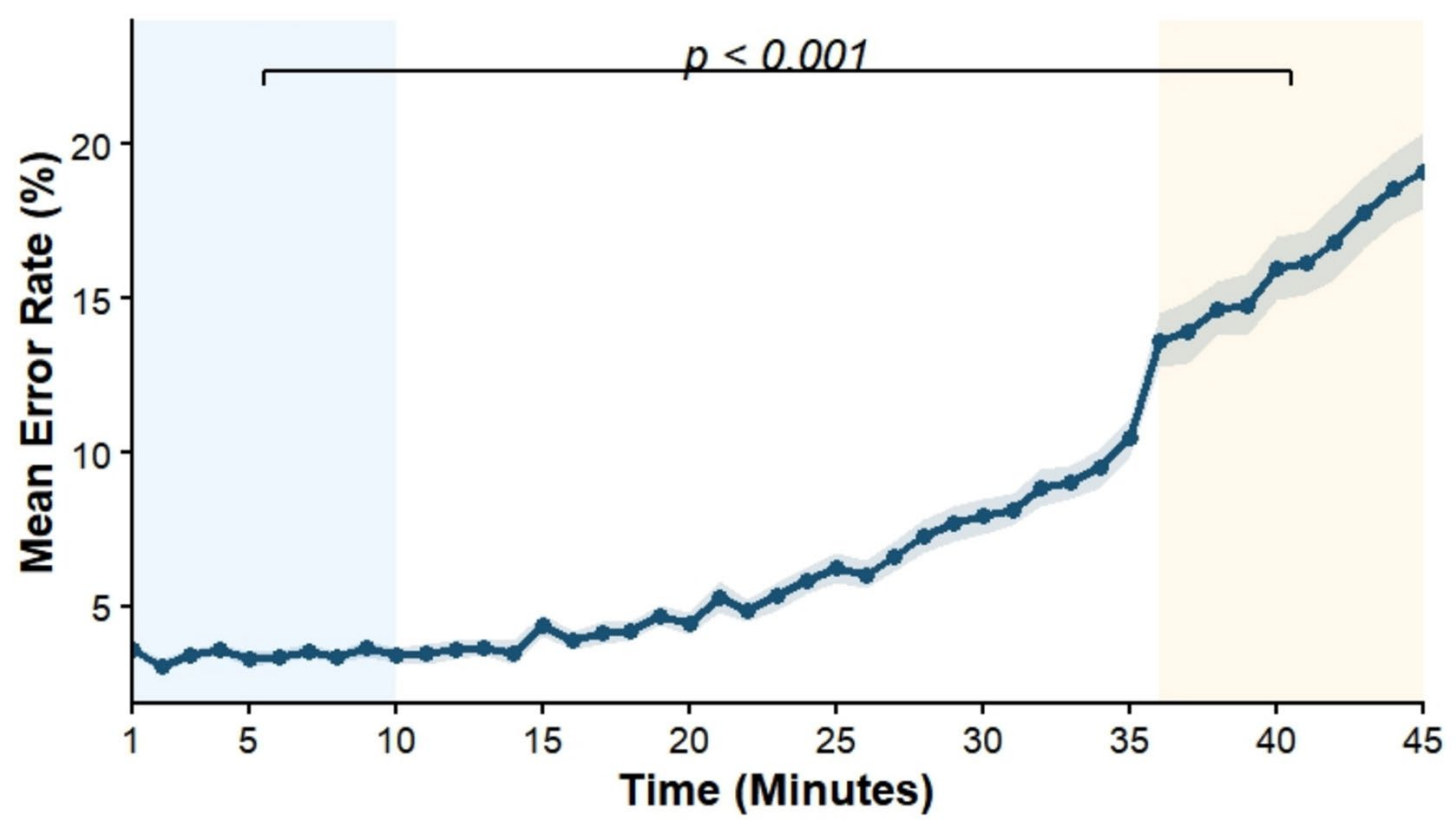
Objective verification of mental fatigue induction via the Time-on-Task effect. The solid line illustrates the continuous change in the mean error rate (%) during the 45-min Stroop color-word conflict task. The shaded band surrounding the mean trajectory represents the standard error of the mean. The light-blue and light-yellow shaded areas denote the first 10 min and the last 10 min of the task, respectively. A statistically significant increase in the mean error rate was observed between these two periods (*p* < 0.001), confirming a severe decline in cognitive execution function by the end of the protocol.

### 3.2 Behavioral outcomes

To evaluate cognitive decision-making and movement execution efficiency under time pressure, temporal variables were compared. The PMT in the MF condition was significantly longer than that in the CON condition (t_(23)_ = 20.874, *p* < 0.001, *d* = 4.26). Conversely, the CCT in the MF condition was significantly shorter than in the CON condition (t_(23)_ = 29.550, *p* < 0.001, *d* = 6.03). This indicates that mental fatigue severely delayed cognitive processing, which subsequently compressed the available time window for actual movement execution (Figure 3).

**Figure 3:**
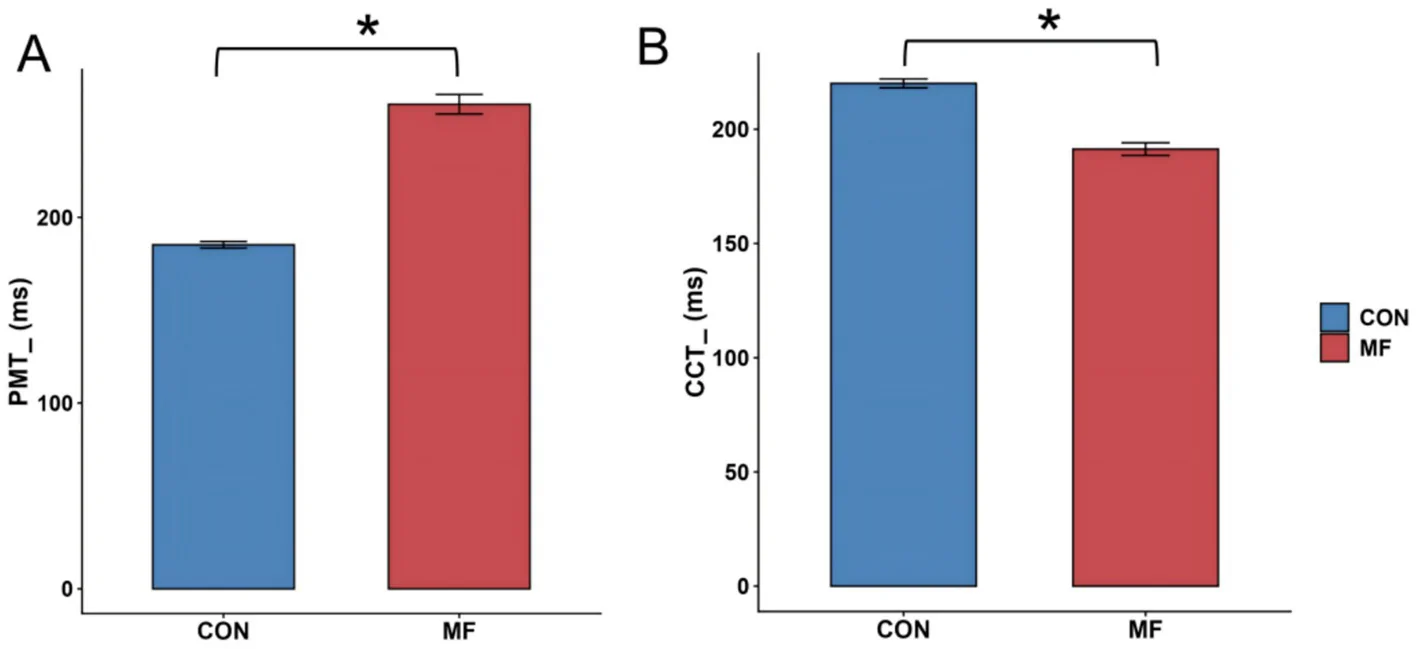
Comparison of behavioral temporal outcomes between the control (CON) and mental fatigue (MF) conditions. **(A)** Premotor time (PMT, ms), reflecting the duration of cognitive decision-making. **(B)** Motor execution time (CCT, ms), reflecting the duration of the physical movement phase. Bar heights represent the mean values, and error bars indicate the standard error of the mean (SEM). Asterisks (*) denote statistically significant differences between the two conditions (*p* < 0.001).

### 3.3 Biomechanical characteristics

During the P1 phase, as shown in Table 1, the maximum ankle dorsiflexion angle in the MF condition was significantly smaller than in the CON condition (t_(23)_ = 2.778, *p* = 0.011, *d* = 0.57). The maximum knee flexion angle did not show a statistically significant difference between the two conditions (*p* = 0.127). Dynamically, the peak vertical GRF (P1_PvGRF) significantly increased in the MF condition compared to the CON condition (t_(23)_ = −3.662, *p* = 0.001, *d* = 0.75). Consistent with this impact amplification, the vertical GRF loading rate (P1_LR) in the MF condition was significantly higher than in the CON condition (t_(23)_ = −4.766, *p* < 0.001, *d* = 0.97). Additionally, the peak knee extension moment was significantly reduced in the MF condition compared to the CON condition (t_(23)_ = 3.276, *p* = 0.003, *d* = 0.67).

**Table 1:** Lower-limb biomechanical characteristics during the initial landing (P1) and post-stroke stabilization (P3) phases across conditions.

Variable	Condition		*df*	*t*	*p*	Cohen’s *d*
	MF (M ± SD)	CON (M ± SD)				
Joint angles (°)						
P1_Knee	58.63 ± 2.10	59.69 ± 4.27	23	1.581	0.127	0.32
P1_Angle	21.21 ± 1.74	21.87 ± 2.26	23	2.778	0.011*	0.57
P3_Knee	43.61 ± 4.43	46.86 ± 2.14	23	3.549	0.002*	0.72
Ground reaction force (GRF)						
P1_vGRF (BW)	1.53 ± 0.28	1.41 ± 0.29	23	−3.662	0.001*	0.75
P1_LR (BW/s)	40.47 ± 8.83	34.68 ± 7.37	23	−4.766	<0.001*	0.97
P3_MLGRF (BW)	0.64 ± 0.03	0.52 ± 0.13	23	−4.574	<0.001*	0.93
Joint moments (Nm/kg)						
P1_Knee	1.53 ± 0.08	1.71 ± 0.26	23	3.276	0.003*	0.67

Values are presented as Mean±Standard Deviation (SD). CON, control condition; MF, mental fatigue condition; df, degrees of freedom; vGRF, vertical ground reaction force; BW, body weight; LR, loading rate; MLGRF, mediolateral ground reaction force. Asterisks (*) indicate a statistically significant difference between the MF and CON conditions (*p* < 0.05). The *p*-values reported in this section reflect uncorrected independent paired *t*-tests. For the strictly conservative Bonferroni-adjusted *p*-values across all biomechanical variables, please refer to Supplementary Table S1.

During the P2 phase, significant decreases were observed in the MF condition for both peak hip extension angular velocity (t_(23)_ = 6.935, *p* < 0.001, *d* = 1.42) and peak knee extension angular velocity (t_(23)_ = 4.601, *p* < 0.001, *d* = 0.94). The peak ankle plantarflexion angular velocity showed no significant difference (*p* = 0.310). For joint kinetics, while the peak hip extension moment did not differ significantly between the two conditions (*p* = 0.152), the MF condition exhibited significantly lower peak knee extension moment (t_(23)_ = 5.272, *p* < 0.001, *d* = 1.08) and peak ankle plantarflexion moment (t_(23)_ = 3.503, *p* = 0.002, *d* = 0.72, Figure 4).

**Figure 4:**
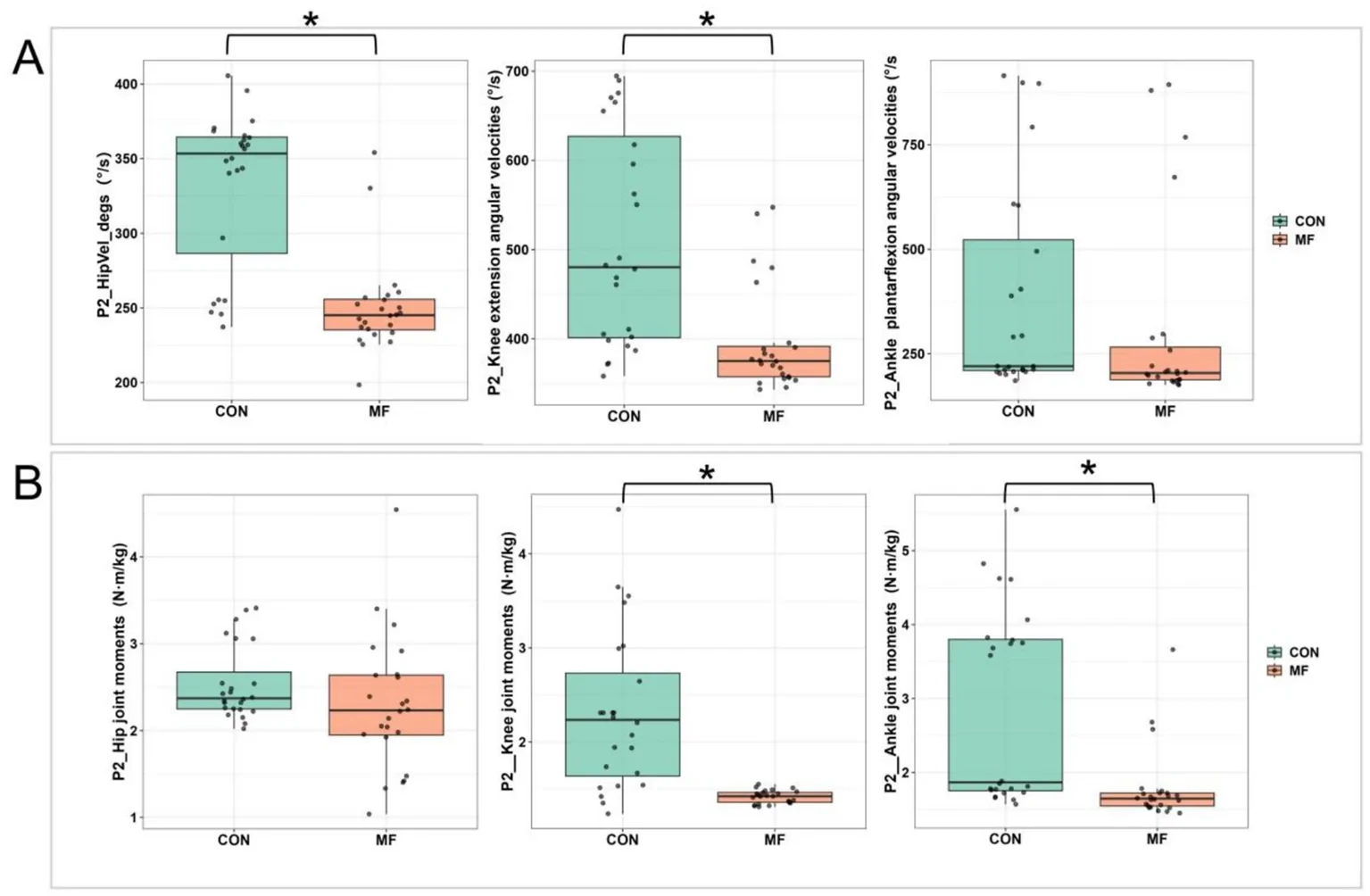
Kinematic and kinetic alterations of the lower-limb joints during the propulsion phase (P2). **(A)** Peak extension/plantarflexion angular velocities (°/s) of the hip, knee, and ankle joints. **(B)** Peak extension/plantarflexion joint moments (Nm/kg) of the hip, knee, and ankle joints. Within each boxplot, the horizontal central line represents the median, and the upper and lower boundaries of the box indicate the interquartile range (25th to 75th percentiles). Individual data points are overlaid to illustrate the data distribution. Asterisks (*) denote statistically significant differences between the control (CON) and mental fatigue (MF) conditions (*p* < 0.05). Specifically, significant reductions under the MF condition were observed for peak hip and knee extension angular velocities **(A)**, as well as peak knee and ankle joint moments **(B)**.

During the P3 phase, the minimum knee flexion angle in the MF condition was significantly smaller than in the CON condition (t_(23)_ = 3.549, *p* = 0.002, *d* = 0.72), indicating a stiffer posture. Most notably, the peak MLGRF in the MF condition was significantly higher than in the CON condition (t_(23)_ = −4.574, *p* < 0.001, *d* = 0.93, Table 1).

## 4 Discussion

### 4.1 Validation of mental fatigue induction

The 45-min Stroop color-word conflict task successfully induced a state of profound MF, evidenced by both objective cognitive decline and subjective workload metrics. The exceptionally large effect sizes observed in both the stroop error rate (*d* = 3.03) and the NASA-TLX scores (*d* = 9.03) align with the theoretical framework of Van Cutsem et al. (2017), regarding the depletion of central cognitive resources. It should be noted that these uncommon magnitudes are partially driven by the mathematical nature of the d formula in within-subject designs, which yields unusually small standard deviations by eliminating inter-individual variance. Furthermore, the extreme effect size in the NASA-TLX may reflect a ceiling effect and the limited sensitivity of the rating scale when assessing profound cognitive exhaustion. Notably, the subjective fatigue reported in our study was slightly higher than that in similar protocols (Zheng et al., 2025b). This heightened fatigue is likely attributable to the demanding nature of the reactive chasse-step task itself, which requires continuous, simultaneous cognitive decision-making and complex motor execution, thereby exacerbating the depletion of central resources. Regarding the experimental design, the control condition involved watching a neutral documentary to maintain wakefulness and prevent confounding boredom-induced fatigue (Van Cutsem et al., 2017). However, as prior research suggests, documentary viewing itself may impose mild cognitive demands (Hachard et al., 2020), meaning it may not represent an absolute zero-cognitive-load baseline. Crucially, this implies that the robust biomechanical impairments observed under the MF condition might actually represent a conservative underestimation of the true magnitude of the MF effect.

### 4.2 The cognitive-motor temporal compression effect

A fundamental finding of this study is the bidirectional “cognitive delay–motor compression” effect associated with MF. Participants exhibited significantly prolonged PMT alongside a shortened CCT. PMT reflects the cognitive processing duration from stimulus presentation to movement initiation. The significant delay in PMT under MF likely stems from compromised executive function in the prefrontal cortex (PFC). While direct neurophysiological evidence was not collected in the present study, prior neuroimaging research provides a speculative mechanism: MF may be associated with glutamate accumulation in the lateral prefrontal cortex, which could disrupt synaptic transmission efficiency and thereby impair executive functions and decision speed (Steward et al., 2025). This is consistent with Englert and Bertrams (2014), who found delayed sprint reaction times under ego depletion, and Gantois et al. (2020), who reported impaired passing decisions in fatigued soccer players. The significant shortening of CCT indicates that, given a restricted total response window, the time available for lower-limb repositioning is severely compromised. Rather than an absolute temporal compression of the entire motor program, this shortening reflects an altered, and potentially hazardous, movement strategy. To compensate for the delayed cognitive processing (i.e., prolonged PMT), athletes may prematurely terminate the chasse-step motion to ensure sufficient time remains for the impending stroke execution. However, this rushed repositioning strategy critically limits the time available for optimal joint shock absorption and kinetic chain preparation, ultimately decreasing mechanical efficiency and potentially elevating injury risks. This aligns with Kong et al. (2023), who noted that MF profoundly alters lower-limb biomechanics during unanticipated side-cutting, suggesting a systemic reorganization of the movement’s temporal structure.

### 4.3 The altered lower-limb biomechanics

The temporal compression associated with MF was accompanied by immediate biomechanical consequences during the P1 phase. Participants exhibited a significantly reduced ankle dorsiflexion angle accompanied by elevated PvGRF and LR. As Hachard et al. (2020) demonstrated, MF compromises postural control by disrupting multisensory integration. When the central processing of proprioceptive feedback is impaired, the CNS tends to reduce the mechanical degrees of freedom it must manage to maintain overall stability. Consequently, the fine motor control of distal joints (e.g., the ankle) is often compromised, predisposing the foot into a relatively plantarflexed, stiff landing position. Biomechanically, this stiffness inherently reduces the overall energy dissipation capacity of the lower extremity, leading to a more direct and harsh transmission of impact forces (Devita and Skelly, 1992). Furthermore, Xu et al. (2020) highlighted that ankle kinematics play a vital role in landing energy dissipation; reduced dorsiflexion limits the overall energy absorption capacity of the lower limb, thereby shifting the load proximally. Unlike Zheng et al. (2025a), who found significant decreases in both ankle and knee flexion during stop-jump and single-leg landings after MF, our study only observed significant changes at the ankle. This discrepancy is likely task-dependent. Stop-jumps require substantial vertical shock absorption primarily via the knee, whereas the chasse-step focuses on horizontal braking and direction change, inherently limiting knee excursion.

We observed a significant reduction in the peak knee extension moment despite no significant change in the knee flexion angle. Under normal circumstances, the quadriceps must generate substantial eccentric moments to absorb impact energy. This paradoxical “angle-moment decoupling” may represent a unique hallmark of MF: the resource-limited CNS may struggle to effectively coordinate the matching relationship between kinematics and kinetics, ultimately exposing passive joint structures to absorb the amplified impact loads.

During the P2 phase, MF selectively impaired proximal joint functions, characterized by significant reductions in both hip and knee extension angular velocities. In high-speed racket sports, hitting power relies heavily on a smooth “proximal-to-distal” kinetic chain. The diminished rotational velocities of the hip and knee indicate a severe disruption in this energy transfer mechanism, substantially degrading movement economy. Interestingly, while peak knee and ankle moments decreased significantly, the peak hip extension moment was maintained. From a biomechanical perspective, this contrasting pattern may partially reflect the differing mechanical demands imposed on the lower-limb joints during the chasse-step. The distal joints (knee and ankle) are generally considered to contribute substantially to rapid horizontal braking and subsequent propulsive actions during high-speed directional movements (Dos’ Santos et al., 2021). As a result, their mechanical output may be more susceptible to the altered neuromuscular state associated with mental fatigue. In contrast, the hip joint is thought to play a comparatively greater role in proximal trunk stabilization and overall postural control during dynamic movement tasks (Kibler et al., 2006). This relatively stabilizing function may have contributed to the preserved hip extension moment observed under the MF condition. Therefore, rather than reflecting a definitive CNS prioritization strategy, the preservation of hip moment may instead indicate a combined effect of task-specific joint mechanical demands and the motor system’s tendency to preserve proximal stability while tolerating reductions in distal joint power output (Zazulak et al., 2007). This shift toward a proximal rigidity–distal weakness pattern explains the decline in explosive performance typically observed in fatigued athletes. Interestingly, while proximal joint velocities decreased significantly, the peak ankle plantarflexion angular velocity remained unaltered under mental fatigue. From a proximal-to-distal kinetic chain perspective, this non-significant finding carries crucial biomechanical implications. It may suggest a distal compensatory mechanism: the ankle joint actively attempts to maintain its propulsive output to counterbalance the significant rotational velocity deficits incurred at the proximal joints. This distal compensation ensures that the overall forward momentum necessary to complete the reactive chasse-step is not entirely compromised.

The most clinically significant findings emerged during the P3 phase. Under MF, participants exhibited a decreased minimum knee flexion angle, reflecting a stiffer, more extended knee posture, alongside a massive increase in MLGRF. Functionally, landing with a knee near full terminal extension minimizes the lower limb’s effective shock-absorbing capacity and limits the dynamic stabilization provided by the surrounding musculature. More critically, the spike in MLGRF directly reflects impaired frontal-plane stability. During rapid cutting, the CNS must continuously process multi-sensory feedback to activate hip abductors and stabilize the knee (Powers, 2010). MF may disrupt this millisecond-level feedback loop, potentially contributing to frontal-plane postural deficits. Biomechanical consensus indicates that knee extension combined with excessive multi-planar loading, specifically frontal-plane shear forces, contributes to non-contact anterior cruciate ligament (ACL) injuries (Kiapour et al., 2016; Hewett et al., 2005). Therefore, the increased MLGRF observed under MF, while not a direct clinical measure, highlights a suboptimal biomechanical risk profile that could potentially elevate lower limb injury susceptibility over time.

Synthesizing the current findings, the impact of mental fatigue on the reactive footwork appears to be closely linked to a cognitive-motor temporal compression. Specifically, mental fatigue delays cognitive processing (evidenced by prolonged PMT), which appears to encroach upon the available time for the subsequent motor program, contributing to a compressed CCT. Under this severe temporal constraint, the lower-limb biomechanical coordination undergoes a systemic reorganization. To cope with the rushed execution, the motor system appears to sacrifice distal joint cushioning to maintain proximal core stability, characterized by restricted ankle dorsiflexion, reduced proximal rotational velocities, and selectively preserved hip extension moments. Consequently, the restricted ankle dorsiflexion and a more extended knee posture facilitate a stiff landing pattern, which is accompanied by significantly higher vertical ground reaction forces and loading rates. Concurrently, this rapid and stiff execution compromises frontal-plane stability—indicated by the elevated MLGRF—subjecting the knee joint to hazardous multi-planar loading.

### 4.4 Limitations

Although this study provides novel insights into the neuro-mechanical coupling during reactive footwork, several methodological limitations must be acknowledged. First, regarding methodological constraints, the exact moment of racket-ball impact was not directly measured. Consequently, all temporal variables were defined exclusively using objective GRF and kinematic data, which provided consistent and reliable event markers across all trials. Specifically, CCT was quantified as the duration to complete the first chasse-step, representing the most critical component of reactive footwork. Additionally, the current study relied on inverse dynamics to infer neuromuscular control strategies. The absence of synchronized electromyography (EMG) data precludes the direct validation of altered muscle pre-activation or co-contraction patterns (e.g., hamstring-to-quadriceps co-activation ratio) during the stiff landing phase.

Second, statistical limitations regarding effect sizes and multiple comparisons must be acknowledged. The exceptionally large effect sizes observed in subjective metrics (d = 9.03 for NASA-TLX) are partially a mathematical artifact of the d_z_ formulation used for within-subject designs, which inflates magnitudes by eliminating inter-individual variance. These extremes may also reflect ceiling effects of the rating scales. Furthermore, to preserve statistical power for exploratory biomechanical profiling, we did not apply familywise error corrections (e.g., Bonferroni) to the multiple independent paired t-tests. While this prevents masking true coordinate adjustments in highly interdependent data, it inevitably inflates the risk of Type I errors.

Finally, sample and contextual constraints should be noted. While the current study maintained a strictly balanced 1:1 sex ratio (12 males and 12 females) to prevent either sex from skewing the overall group means, the statistical analysis evaluated the universal biomechanical response to mental fatigue. Due to sample size constraints, we did not conduct sex-stratified analyses or utilize mixed-effects models to adjust for sex covariates. This is a notable limitation, as biological sex can act as a significant confounding factor. Females may exhibit inherent biomechanical differences, such as greater knee valgus, lower baseline joint stiffness, and distinct neuromuscular activation strategies compared to males (Chappell et al., 2002). Consequently, the universal biomechanical response to mental fatigue reported here may mask sex-specific compensatory mechanisms.

### 4.5 Future directions

Based on the identified limitations and the key findings of the current study, future research should pursue both methodological improvements and broader theoretical expansions. From a method-improvement perspective, future confirmatory studies should integrate synchronized racket-sensor data to capture the entire stroke cycle, and employ synchronized EMG to elucidate specific neuromuscular mechanisms. Furthermore, employing advanced multivariate frameworks, such as statistical parametric mapping (SPM) or linear mixed-effects models, is recommended to holistically evaluate continuous kinematic synergies while rigorously controlling for familywise error rates and accounting for biological variables like sex.

From an expansion-oriented perspective, our findings regarding the cognitive-motor time compression effect open several forward-looking avenues. First, future studies should shift from analyzing isolated local temporal variables to employing whole-movement dynamic coordination network analyses, mapping the systemic reorganization of the kinetic chain under fatigue. Second, rather than solely focusing on group-level mean comparisons, investigating inter-individual differences is warranted to identify specific adaptive strategies and determine which athletes are most mechanically susceptible to cognitive fatigue. Finally, future research should transition from isolated acute reactive tasks to complex, dual-task interference paradigms, thereby achieving higher ecological validity and providing deeper insights into how athletes balance performance and injury prevention under authentic competitive stress.

### 4.6 Practical applications

The findings of this study offer crucial, evidence-based insights for injury prevention and performance optimization in racket sports. Primarily, coaches and athletic trainers must recognize neurocognitive fatigue as a potent precursor to biomechanical breakdown. High-risk, high-intensity reactive agility or cutting drills should be strictly avoided immediately following cognitively demanding tasks, such as intensive tactical video analysis or academic exams for collegiate athletes. Furthermore, injury prevention programs for fatigued athletes should prioritize real-time biomechanical feedback, utilizing concrete monitoring thresholds. For example, practitioners could employ portable force plates or 2D video analysis to establish an athlete’s rested baseline; a decline in maximum ankle dorsiflexion exceeding 10%–15%, or a spike in vertical loading rates of over 15% during landing, should serve as an actionable threshold to terminate high-risk reactive drills. Strengthening the hip abductors to enhance frontal-plane stability could also mitigate the dangerous spikes in MLGRF observed under fatigue. Finally, integrating subjective monitoring tools, such as the NASA-TLX, into daily routines, alongside specialized cognitive-motor dual-task training, may effectively bolster an athlete’s neuro-mechanical resilience against the detriments of mental fatigue.

## 5 Conclusion

This study investigated the influence of mental fatigue on the biomechanical control of the reactive chasse-step in table tennis athletes. The results demonstrate that mental fatigue is associated with a specific reorganization of the cognitive-motor temporal structure, characterized by a prolonged PMT and a concurrently shortened CCT. This observed temporal compression appears to be a primary factor associated with subsequent alterations in lower-limb biomechanics. In the P1 phase, MF was characterized by a stiff landing pattern, evidenced by restricted ankle dorsiflexion and an increase in both vertical ground reaction forces and loading rates. This altered coordination continued into the P2 phase, where reductions in hip and knee extension velocities, along with diminished knee and ankle moments, suggested a compromise in kinetic chain efficiency. Furthermore, during the P3 phase, participants exhibited a more extended knee posture and a significant increase in MLGRF. These biomechanical shifts suggest that MF may not only impair athletic performance but also expose athletes to suboptimal joint loading patterns. Such altered mechanics are widely recognized as potential biomechanical risk factors for non-contact lower-limb injuries, highlighting the need for fatigue monitoring in reactive sporting environments. In conclusion, the cognitive state of an athlete appears to be a relevant factor in maintaining stable and efficient reactive motor control. These findings suggest that practitioners should consider the impact of cognitive load when monitoring training and may benefit from avoiding high-pressure reactive agility drills when athletes exhibit signs of significant mental fatigue to better balance performance and injury prevention.

## Data Availability

The original contributions presented in the study are included in the article/Supplementary material, further inquiries can be directed to the corresponding authors.
