# Supplementary material for: Delayed decision, harder landing: how mental fatigue alters reactive step biomechanics and joint loading in table tennis players
Source: Front Psychol. 2026 Oct 1;17:1871714. doi: 10.3389/fpsyg.2026.1871714 (PMC13671105; doi:10.3389/fpsyg.2026.1871714)
Supplement: Supplementary file 1 [file Table_1.docx]

Table S1. Original and Bonferroni-adjusted *p*-values for the lower-limb biomechanical variables across the reactive chasse-step phases.

| **Biomechanical Variable** | **Original *p*-value** | **Bonferroni-adjusted *p*-value** | **Adjusted Significance** |
| --- | --- | --- | --- |
| **Phase 1 (P1)** |  |  |  |
| Peak knee flexion angle (°) | 0.127 | 1.000 | *ns* |
| Peak ankle dorsiflexion angle (°) | 0.011 | 0.143 | *ns* |
| Peak vertical GRF (BW) | 0.001 | 0.013 | *p* < 0.05 |
| Vertical loading rate (BW/s) | < 0.001 | < 0.013 | *p* < 0.05 |
| Peak knee extension moment (N·m/kg) | 0.003 | 0.039 | *p* < 0.05 |
| **Phase 2 (P2)** |  |  |  |
| Peak hip extension angular velocity (°/s) | < 0.001 | < 0.013 | *p* < 0.05 |
| Peak knee extension angular velocity (°/s) | < 0.001 | < 0.013 | *p* < 0.05 |
| Peak ankle plantarflexion angular velocity (°/s) | 0.310 | 1.000 | *ns* |
| Peak hip extension moment (N·m/kg) | 0.152 | 1.000 | *ns* |
| Peak knee extension moment (N·m/kg) | < 0.001 | < 0.013 | *p* < 0.05 |
| Peak ankle plantarflexion moment (N·m/kg) | 0.002 | 0.026 | *p* < 0.05 |
| **Phase 3 (P3)** |  |  |  |
| Minimum knee flexion angle (°) | 0.002 | 0.026 | *p* < 0.05 |
| Peak medial-lateral GRF (BW) | < 0.001 | < 0.013 | *p* < 0.05 |

Notes: The multiple comparison corrections were specifically applied to the family of exploratory biomechanical variables (n = 13) extracted across the three functional phases. Behavioral and fatigue-manipulation metrics (e.g., NASA-TLX, PMT) were considered distinct conceptual domains and were not included in this biomechanical familywise correction.
